# INTERVERTEBRAL DISC DEGENERATION INSTIGATES VERTEBRAL ENDPLATE REMODELING AND FACET JOINT PATHOLOGY IN A LARGE ANIMAL MODEL

**DOI:** 10.22203/ecm.v047a09

**Published:** 2024-04-23

**Authors:** S.E. Gullbrand, B.S. Orozco, M. Fainor, K. Meadows, R. Hilliard, M. Boyes, S. Mahindroo, R.L. Mauck, D.M. Elliott, T.P. Schaer, H.E. Smith

**Affiliations:** 1 Department of Orthopaedic Surgery, McKay Orthopaedic Research Laboratory, Perelman School of Medicine, University of Pennsylvania, 19104 Philadelphia, PA, USA; 2 Translational Musculoskeletal Research Center, Corporal Michael J. Crescenz VA Medical Center, 19104 Philadelphia, PA, USA; 3 Department of Biomedical Engineering, University of Delaware, 19716 Newark, DE, USA; 4 Department of Clinical Studies, New Bolton Center, School of Veterinary Medicine, University of Pennsylvania, 19104 Philadelphia, PA, USA; 5 Department of Bioengineering, University of Pennsylvania, 19104 Philadelphia, PA, USA

**Keywords:** Goat model, crosstalk, osteoarthritis, disc nutrition

## Abstract

Although the intervertebral discs are the most studied region of the spinal motion segment with respect to their degeneration and contributions to back pain, it is becoming increasingly evident that degeneration of adjacent structures including the facet joints, vertebral endplates and paraspinal muscles occurs concomitant with disc degeneration. However, crosstalk between these adjacent components of the motion segment remains understudied, particularly in preclinical large animal models. In this study, intervertebral disc degeneration was induced in goat cervical discs via intradiscal injection of 2U or 5U of chondroitinase ABC (ChABC). Disc degeneration and trans-endplate small molecule diffusion into the disc were assessed at 12 weeks using *in vivo* MRI T2 mapping and post-contrast enhanced T1 mapping. Animals were euthanized at 12 weeks post-ChABC injection for end-term structure-function analysis of the disc, vertebral endplate and facet tissues. Intradiscal injection of ChABC yielded a spectrum of disc degeneration independent of ChABC dosage. Increasing severity of disc degeneration correlated with increased vertebral endplate bone density. In levels that did not exhibit severe degeneration or endplate resorptions, we demonstrated a significant correlation between NP T2 values and disc diffusion properties. Early-stage osteoarthritis of the facet joints was observed concomitant with disc degeneration, characterized primarily by alterations in facet cartilage mechanical properties. This work established a large animal model of whole spinal motion segment degeneration, including correlations between trans-endplate diffusion and disc health, which can be utilized to increase the translational relevance of studies evaluating strategies for disc regeneration or repair.

## Introduction

Globally, low back pain is the number one cause of disability, and back pain is the second most common reason for a doctor’s visit after the common cold (BCC Research Staff, 2018). In the United States, back pain is the number one condition contributing to healthcare spending, totaling $134.5 billion in 2016 alone (Dieleman *et al*., 2020). The basic unit of the spine is termed the motion segment. The anterior compartment of the motion segment consists of the intervertebral disc and superior and inferior cartilage endplates (CEP) and bony vertebral bodies. Intervertebral discs are essential for spine mechanical function, and are composed of a central, proteoglycan and water rich nucleus pulposus (NP), surrounded by a lamellar, collagenous annulus fibrosus (AF). Posteriorly lies the spinal cord (or cauda equina, depending on spinal level) and nerve roots, a pair of diarthrodial facet joints, and the paraspinal muscles (Ashinsky *et al*., 2021). While the causes of back pain are complex and can include psychosocial factors, these anatomic structures within the spine are considered direct contributors to back pain – particularly degenerative pathology within the intervertebral disc, the facet joints and the vertebral end-plates (Hartvigsen *et al*., 2018). Intervertebral disc degeneration is the most commonly studied contributor to back pain. Disc degeneration is a cascade of cellular, compositional, structural and mechanical alterations to the AF and NP tissues (Haefeli *et al*., 2006). The degenerative disc can be a direct source of pain, or degeneration can culminate in a loss of disc height, causing the disc to bulge and impinge upon the adjacent neural elements. Due to the biological and mechanical interplay that occurs between the disc, end-plates and facet joints, there is a growing research interest in studying crosstalk between the disc and these other spinal substructures (Ashinsky *et al*., 2021; Fine *et al*., 2023).

As the intervertebral discs are the largest avascular tissues in the body, the endplates play a critical role in disc homeostasis (Ashinsky *et al*., 2021). Nutrients and waste products must travel from the vascular buds that terminate in the bony endplate, through the cartilaginous endplate, and into the disc (Nachemson *et al*., 1970). Degeneration of the cartilaginous EP and vertebral EP is often observed concomitant with disc degeneration. Endplate lesions (Schmorl’s nodes) have been identified on magnetic resonance imaging (MRI) and histologic sections of human disc tissue, (Hilton *et al*., 1976) and the presence of endplate lesions is associated with back pain, local inflammation and innervation (Wang *et al*., 2012). Cartilage endplate defects are strong predictors of chronic low back pain, even after adjusting for the presence of Modic changes and disc degeneration (Bailey *et al*., 2019).

Because of the inherent mechanical linkage between the disc and facet joints, the spinal motion segment is often referred to as a three joint complex (Yong-Hing and Kirkaldy-Willis, 1983). The facets connect adjacent spinous processes posteriorly and bear 2–25 % of the axial loads through the spine, in a healthy state and depending on spinal level, which can increase to up to 70 % with severe loss of disc height due to degeneration (Jaumard *et al*., 2011; Yang and King, 1984). The facet joints also impart torsional stiffness in addition to resistance to shear, lateral and antero-posterior vertebral translation, and joint distraction (Jaumard *et al*., 2011), (Raynor *et al*., 1985). Osteoarthritis (OA) of the spinal facet joints, which is very prevalent in older adults and frequently observed concomitant with disc degeneration, is implicated as a contributor to back pain in 20 to 50 % of patients, depending on age (Gellhorn *et al*., 2013).

The clinical treatment of back pain is limited to physical therapy, pain management (oral pain medication, steroid injections, nerve blocks), or invasive surgical procedures (such as spinal fusion) which do not restore healthy spine structure or function. Because of this, there continues to be substantial research efforts towards developing tissue engineering and regenerative medicine approaches for spinal degenerative pathology to treat individuals with back pain (DiStefano *et al*., 2022; Gullbrand *et al*., 2018; Panebianco *et al*., 2020; Sakai and Andersson, 2015). Successful translation of these emerging technologies will require preclinical animal models which replicate the spectrum of human disease. A wide variety of animal models of intervertebral disc degeneration have been established across multiple species, including mice (Melgoza *et al*., 2021), rats (Lai *et al*., 2021), rabbits (Gullbrand *et al*., 2021), sheep, goats, and dogs (Lee *et al*., 2021). Advantages of large animal models include their relative geometry, biomechanics and comparative anatomy to the human spine (Lee *et al*., 2021). Although naturally occurring disc degeneration does occur in some species, disc degeneration is often experimentally induced via either disc injury, altered mechanical loading, or enzymatic NP degradation (chemonucleolysis). Chondroitinase ABC (ChABC) is commonly used for chemonucleolysis, and specifically degrades the chondroitin and dermatan sulfate side chains of proteoglycans, thereby recapitulating the hallmark loss of proteoglycans which occurs in early-stage human degeneration (Hoogendoorn *et al*., 2007).

Given that, in humans, degeneration is rarely present in only one spinal structure, there is a need to characterize animal models where degeneration occurs across the whole motion segment to increase the translational relevance of future studies testing the efficacy of tissue engineering or regenerative medicine approaches. Our previous work using the rabbit disc puncture model established that end-plate remodeling during disc degeneration reduces small molecule diffusion into the disc (Ashinsky *et al*., 2020), however the anterior bridging osteophytes which occur in that model limited our study of disc-facet joint mechanical crosstalk (Fainor *et al*., 2023). Large animal models of disc degeneration have thus far focused only on establishing and assessing degeneration to the disc tissues, without consideration of concomitant pathology in the endplates or facet joints, and have primarily focused on the lumbar spine (Borem *et al*., 2021; Gullbrand *et al*., 2017). The goat cervical spine can serve as model for both the human cervical and lumbar spine, and is an ideal model in which to study crosstalk in the spinal motion segment. Nutritional constraints to the disc in humans are matched in goats due to the large size of the cervical discs, and the semi-upright nature (compared to the horizontal lumbar spines of quadrupeds) and comparable intradiscal pressures to the human spine facilitate the study of disc-facet mechanical crosstalk during degeneration (Reitmaier *et al*., 2013). The purpose of this study was therefore to investigate concomitant pathology in the boney endplates and facet joints in goat cervical spine model of induced disc degeneration, and to determine correlations between loss of disc health and these adjacent structures in the motion segment.

## Methods

### Animal Surgical Procedure and Study Overview

The study was approved by the Institutional Animal Care and Use Committee of the University of Pennsylvania (Protocol number 805699). Eight large frame goats (castrated males), approximately 2–5 years of age (73.7 ± 13.3 kg), were used in this study (Thomas D. Morris, Inc. Reisterstown, MD, USA). Animals were group housed with unrestricted exercise in a barn with natural bedding for the duration of the study. Throughout the study, animals were assessed daily for clinical signs of pain or distress by a veterinarian.

**Fig. 1.**
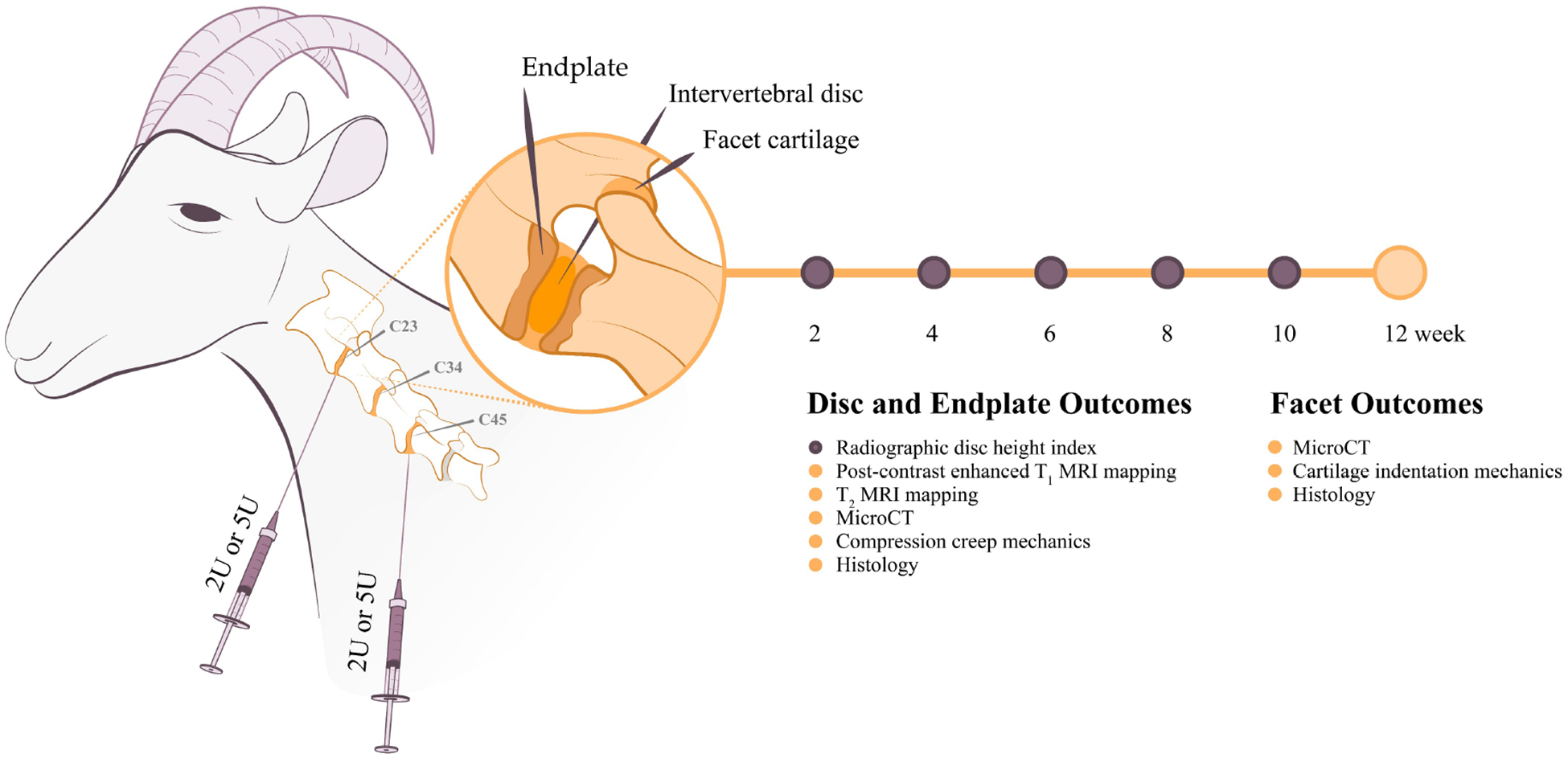
Study overview. Disc degeneration was induced at the C2-C3 and C4-C5 levels of the goat cervical spine via injection of either 2U or 5U ChABC. Each animal received both doses of ChABC, randomized across the C2-C3 and C4-C5 levels. The intervening C3-C4 disc was utilized as a healthy control. Radiographs were obtained every 2 weeks to measure the disc height index. *In vivo* MRIs were obtained at 12 weeks post-ChABC. Following euthanasia, each spinal level was separated into the anterior column (vertebral body – disc – vertebral body) and posterior facet joints for the indicated analyses.

All animals underwent a surgical procedure to induce degeneration of the cervical intervertebral discs, as summarized in Fig. 1. Animals were sedated with diazepam (0.5–1.5 mg/kg, intravenous [IV]/intramuscular [IM]) or midazolam (0.3–05 mg/kg IV/IM) followed by induction for general anesthesia with ketamine (2.2–4.0 mg/kg, IV) and maintained on inhalation anesthesia using 1–5 % Isoflurane in oxygen. Animals were positioned in dorsal recumbency and fluoroscopy was utilized to identify the C2-C3, C3-C4 and C4-C5 disc spaces. A 6-inch 22G spinal needle was inserted at the level of the ventral intervertebral disc into the nucleus pulposus of the C2-C3 and C4-C5 disc spaces via a percutaneous approach under fluoroscopic control (Arcadis Orbis, Siemens, Munich, Germany). 200μL of either 2U or 5U chondroitinase ABC (ChABC, Amsbio, Cambridge, MA, USA) suspended in 200 μL of buffer (sterile phosphate buffered saline (PBS)) containing 0.1 % bovine serum albumin) were injected. These dosages were selected based on our prior work utilizing ChABC in the goat lumbar disc (which are smaller than the cervical discs). Our goal here was to achieve a more degenerative pathology to create a model in which to evaluate a whole, tissue engineered disc replacement in future studies (Gullbrand *et al*., 2017; Gullbrand *et al*., 2018). Each animal received both doses of ChABC, with the 2U and 5U injections randomized between the C2-C3 and C4-C5 levels. The C3-C4 level was utilized as an unperturbed control. Our prior work using this model in the lumbar spine has demonstrated that a 200μL injection of buffer alone via the 22G spinal needle yielded no detectable degenerative changes to the discs (Gullbrand *et al*., 2017). Additionally, the ratio of needle diameter to disc height for a 22G needle in the average goat cervical disc is <25 %, which literature suggests should have no detectable effects on the progression of disc degeneration (Elliott *et al*., 2008). For these reasons, a sham injection control was not included in the current study to reduce animal usage. Study animals received buprenorphine (0.005–0.01 mg/kg IV or IM) perioperatively (SID-QID), and/or fentanyl (2.5 mcg/kg/hr transdermal) removed after 72 hours and flunixin meglumine (1.1 mg/kg IV or IM) SID for 3 days beginning the day of surgery. All animals were examined twice daily by veterinarian for signs of neurologic deficits, gait, and clinical well-being for the duration of the study.

The *in vivo* and post-mortem analyses are summarized in Fig. 1 and were performed by individuals blinded to experimental group. *In vivo* analyses included serial lateral radiographs of the cervical spine in the awake and standing animal and cervical spine MRIs at 12 weeks post-ChABC under general anesthesia using a 3T MRI (Siemens 3T Magnetom Prisma, Siemens Healthineers, Erlangen, Germany). Following MRI at the 12-week timepoint, 7 animals were euthanized with an overdose of a commercially available euthanasia solution (Pentobarbital 1 mL/5kg) according to the guidelines set forth by the current AVMA Panel on Euthanasia. One animal was enrolled in an alternate pilot study at 12 weeks post-ChABC and was not included in post-mortem analyses after *in vivo* radiographs and MRI. Following euthanasia, the cervical spines were collected and the C2-C3, C3-C4 and C4-C5 spinal motion segments isolated and separated into the anterior column (vertebral body – intervertebral disc – vertebral body) and the two posterior facet joints (each consisting of superior and inferior articular surfaces) for subsequent structure-function analyses, as detailed below. Isolated anterior motion segments and the facet joints were stored wrapped in saline soaked gauze at − 20 °C until analysis.

### In Vivo Radiographs and Magnetic Resonance Imaging (MRI)

Lateral plain radiographs of the cervical spine were obtained pre-operatively and every two weeks following ChABC injection, in standing, awake animals. A custom MATLAB code was utilized to calculate disc height index (disc height divided by average adjacent vertebra length) at the C2-C3, C3-C4 and C4-C5 level at each time point, according to our established methods (Gullbrand *et al*., 2017; Martin *et al*., 2015). Disc height index (DHI) values were normalized to pre-operative values for each disc.

MRIs of the cervical spine were obtained, under general anesthesia, at 12 weeks post-operatively. A T2 weighted CPMG sequence (TR = 3000 ms, TE = 13.6, 27.2, … 340 ms, in-plane resolution of 0.56 mm × 0.56 mm, FoV 325 mm, slice thickness 5.0 mm) was used to measure T2 relaxation time. A T1-weighted inversion recovery sequence (TR = 15 ms, TE = 2.11 ms, in-plane resolution of 0.42 mm × 0.42 mm, FoV 325 mm, slice thickness 5.0 mm, flip angles = 5 and 26 degrees) was used to measure T1 relaxation time before, and 30 minutes after, injection of gadodiamide contrast agent. Gadodiamide (Omniscan [GE Healthcare, Piscataway, NJ, USA], MW = 573) is a non-protein-bound, non-ionic contrast agent which was delivered intravenously at 0.1 mmol/kg. T2 relaxation time and T1 relaxation time before and after gadodiamide injection were assessed in the NP region in the mid sagittal plane for each disc. The T2 times were analyzed by fitting the intensity in the nucleus pulposus to noise-corrected exponentials (Meadows *et al*., 2020). The T1 times were quantified in ImageJ from the T1 maps, and the % reduction in T1 from pre- to post- gadodiamide injection was calculated as a measure of the uptake of gadodiamide into the disc, where a higher % reduction indicated more diffusion (Ashinsky *et al*., 2020).

### Intervertebral Disc Biomechanical Testing

Motion segments (bone-disc-bone, without facets) underwent compressive mechanical testing using an Instron 5948 to quantify disc compressive mechanical properties, according to our established methods (Gullbrand *et al*., 2017; Gullbrand *et al*., 2018; Martin *et al*., 2017). Briefly, the cranial and caudal vertebral bodies were potted in a low melting temperature alloy, and specimens were subjected to 20 cycles of compression loading from – 0.5 N to – 100 N, followed by a 1 hour creep load at – 100N (~ 0.24 MPa). The compressive load applied is within the range of *in vivo* loading within the goat and human cervical spine (Peterson *et al*., 2018; Pospiech *et al*., 1999). Mechanical testing was conducted in a PBS bath at room temperature. To measure axial displacement during testing, two ink marks were placed on each vertebral body adjacent to the disc, and optically tracked using a digital camera. Force and displacement data were normalized to stress and strain by manually contouring the disc in sagittal and axial MR images to determine disc area and height. Disc area was calculated from the number of pixels within the disc multiplied by in-plane MRI resolution. Disc height was determined by dividing the area of the disc in the midsagittal plan divided by the width of the disc in the anterior-posterior direction. Stress was normalized as force divided by area; strain was normalized as displacement divided by original disc height. A bilinear fit of the 20th cycle of compression was performed using the Bilinear Fit function in MATLAB to identify the toe and linear regions to quantify toe modulus, linear modulus as well as transition strain (defined by the intersection of the two fit lines) and maximal strain.

### Facet Articular Cartilage Biomechanical Testing

Creep indentation testing of the cartilage from one pair of superior and inferior facet articular surfaces per spinal level was performed to quantify changes in facet cartilage properties. To do so, the facet subchondral bone was potted using a low melting temperature alloy. A custom indentation rig coupled with an Instron 5948 electromechanical test frame was used to apply a – 0.1 N creep load for 15 minutes to the facet cartilage through a 2 mm diameter spherical indenter (Gupta *et al*., 2022). Indentation testing was conducted in a PBS bath. Displacement versus time data was fit to a Hertzian biphasic creep model to obtain cartilage compressive modulus, tensile modulus, and hydraulic permeability (Moore *et al*., 2016). Data from three points along the superior and inferior articular surface (6 points per facet joint) were averaged, and then normalized to the average values obtained for the C3-C4 control level. Any indentation tests with an R^2^ value of less than 0.95 to the model fit were excluded from analysis.

### Microcomputed Tomography (μCT)

Following mechanical testing, motion segments and facet articular surfaces (paired superior and inferior surfaces) were fixed in 10 % neutral buffered formalin at 4 °C for 1 week. Motion segments and facet articular surfaces underwent μCT scanning (Scanco μCT50, Brütisellen, Switzerland) at an isotropic 24.2 μm and 10.3 μm resolution, respectively (70 kVp, 114 mA, 250 second integration time). Subchondral bone morphometry was analyzed in the cranial and caudal vertebral endplates and each facet articular surface using Scanco software. The vertebral endplate region of interest (ROI) analyzed consisted of the entire area of the vertebral endplate bounded by the inter-vertebral disc and growth plate and inclusive of any areas of radiolucency caused by bony resorption. Endplate resorptions were noted as present or absent in each level and defined as any region of radiolucency within the defined ROI. The ROI analyzed for each facet articular surface extended 2 mm deep into the subchondral bone, spanning the central 600 slices of each scan.

### Histology

Following μCT scanning, anterior motion segments and one pair of superior and inferior facet joints were decalcified (Formical 2000; Decal Chemical Corporation, Tall-man, USA) and processed into paraffin. Mid-sagittal sections of motion segments were stained with Alcian blue and Picrosirius red (for proteoglycans and collagen, respectively), Hematoxylin and Eosin (to visualize cell morphology and number) or with RGB trichrome (for high contrast staining of bone and cartilage) (Gaytan *et al*., 2020). Mid-sagittal sections of each facet articular surface were stained with Safranin-O and Fast Green (for proteoglycans and collagen, respectively). All stained slides were scanned at 20X magnification using an Aperio slide scanner (Leica Biosystems, Buffalo Grove, IL, USA). Motion segment and facet histology grading were performed by 3 observers (SEG – 10+ years of experience in histology grading, BSO & MF – 2+ years of experience in histology grading) blinded to treatment, and a consensus score was reached. Motion sections were graded using the JOR Spine/ORS Spine Section scoring system for large animals (Lee *et al*., 2021); facet sections were graded using the OARSI histopathology grading system for goats (Little *et al*., 2010).

### Statistical Analyses

Statistical analyses were conducted in Prism 9 (Graph-Pad Software, Boston, MA, USA). Data are shown as mean with standard deviation. Normality of continuous variables was confirmed using the Shapiro-Wilk test. For disc height index measurements, a mixed-effects model was utilized to determine differences between experimental groups (control, 2U or 5U ChABC) at each time point that radiographs were obtained post-ChABC. For all other variables, statistical differences between groups were determined via one-factor ANOVA with Tukey’s multiple comparison test or Dunn’s multiple comparisons test for parametric and non-parametric data, respectively. A Pearson correlation matrix was generated to test for correlations between disc (NP T2 relaxation time, disc histology score, disc mechanical properties) and facet outcomes (facet cartilage mechanical properties, facet OARSI score) across all experimental groups. Statistical significance was defined as *p* < 0.05.

## Results

### Intradiscal ChABC Delivery Instigates Disc Degeneration in the Cervical Spine

**Fig. 2.**
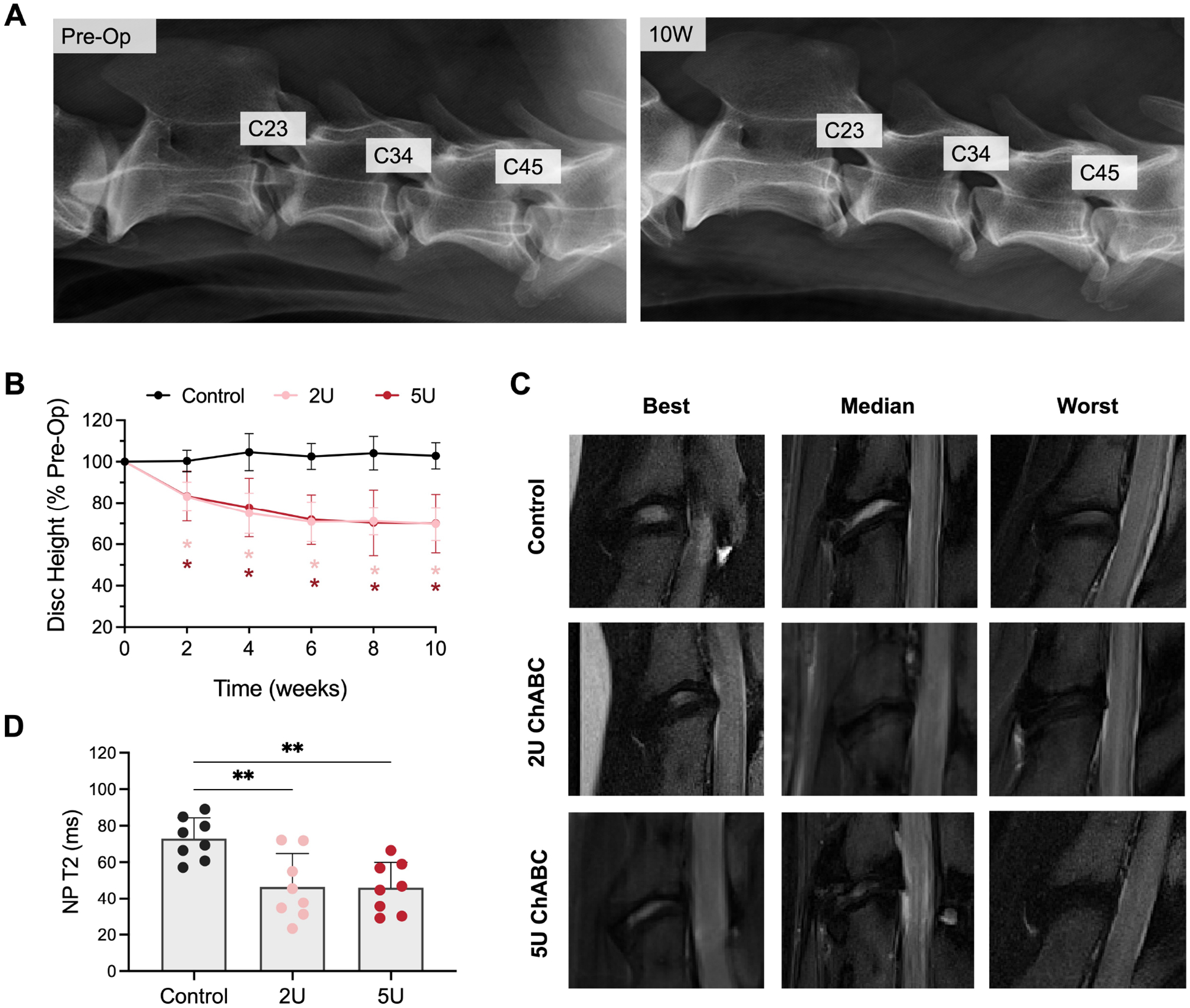
*In vivo* imaging outcomes. (**A**). Representative lateral plain radiographs obtained in standing animals pre-operatively and at 12 week post-ChABC. The C3-C4 disc is the healthy control level, which received no ChABC injection. (**B**) Disc height index measured from radiographs, normalized to pre-operative disc height. * = *p* < 0.05 compared to control. (**C**) Best (least degenerative), median and worst (most degenerative) T2-weighted MRIs from each experimental group. (**D**) Quantification of T2 relaxation times in the NP in each experimental group. ** = *p* < 0.01, n = 8 per group.

Radiographic analysis of disc height index demonstrated a reduction in disc height in discs receiving either 2U or 5U ChABC, which was statistically different from control discs beginning at 2 weeks post-ChABC injection (Fig. 2A,B). At 10 weeks, disc height index was 69.8 % and 70.0 % of pre-operative values in the 2U and 5U groups, respectively. No detectable difference was observed in disc height between the 2U and 5U groups at any time point. *In vivo* T2-weighted MRIs at the 12-week endpoint demonstrated a spectrum of disc degeneration across all experimental groups, characterized by reductions in disc height and NP signal intensity (Fig. 2C). T2 relaxation times in the NP were also calculated by MRI T2 mapping and demonstrated a statistically significant 36.4 % and 36.8 % mean reduction in NP T2 values compared to controls in the 2U and 5U ChABC groups, respectively (Fig. 2D).

**Fig. 3.**
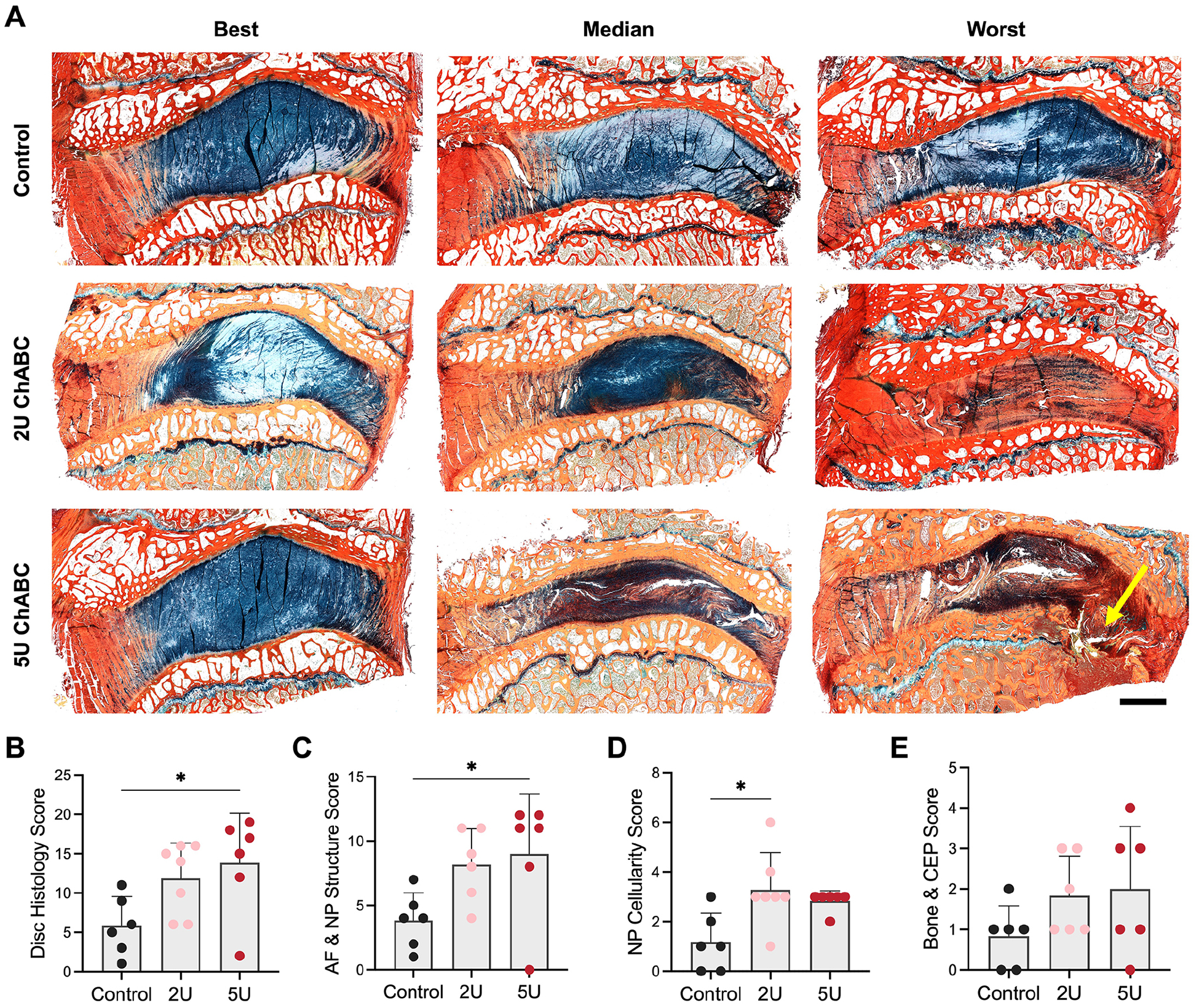
Intervertebral disc histology. (**A**) Best (least degenerative), median and worst (most degenerative) sagittal histology sections stained with Alcian blue and picrosirius red for each experimental group. Scale = 3mm, the yellow arrow denotes the location of an endplate resorption. (**B**) Total disc histology score, which is comprised of a sum of the (**C**) AF and NP structure score (sum of scores for NP matrix staining, AF morphology, N/AF clefts and distinction between the NP/AF subcategories), (**D**) NP cellularity score (sum of scores for NP cell clusters and NP cell loss and necrosis subcategories) and (**E**) bone and cartilage endplate (CEP) score (sum of scores for bone formation and CEP morphology subcategories). A higher score is indicative of a more degenerative disc. * = *p* < 0.05 between groups, n = 6 per group.

Alcian blue and picrosirius red stained sagittal histology sections from each experimental group also highlighted the heterogeneity of disc degeneration at 12 weeks following ChABC delivery (Fig. 3A). Mild to moderate degenerative changes in this model were characterized by initial losses in proteoglycan content in the NP region, along with disorganization of the AF layers. In discs with the most severe degeneration, little proteoglycan staining was present in the NP, the AF was disorganized, and bony endplate defects akin to Schmorl’s nodes were noted. Scoring of disc histology revealed significantly increased scores (total of all scoring categories) in the 5U group compared to controls (Fig. 3B). Mild degenerative changes were also noted in the control group, causing notable overlap in the histology scores between the 2U and control groups. The AF and NP structure sub-score, which sums the categories of NP matrix staining, presence of NP/AF clefts, AF morphology and distinction between the NP and AF, was significantly increased in the 5U ChABC group compared to controls (Fig. 3C). The NP cellularity sub-score, which sums the categories of NP cell clusters and NP cell loss/necrosis, was significantly increased in the 2U ChABC group compared to controls (Fig. 3D). There were no detectable differences in the Bone and CEP sub-score across groups (Fig. 3E).

### Stratification of Samples for Analysis by NP T2 Values

**Fig. 4.**
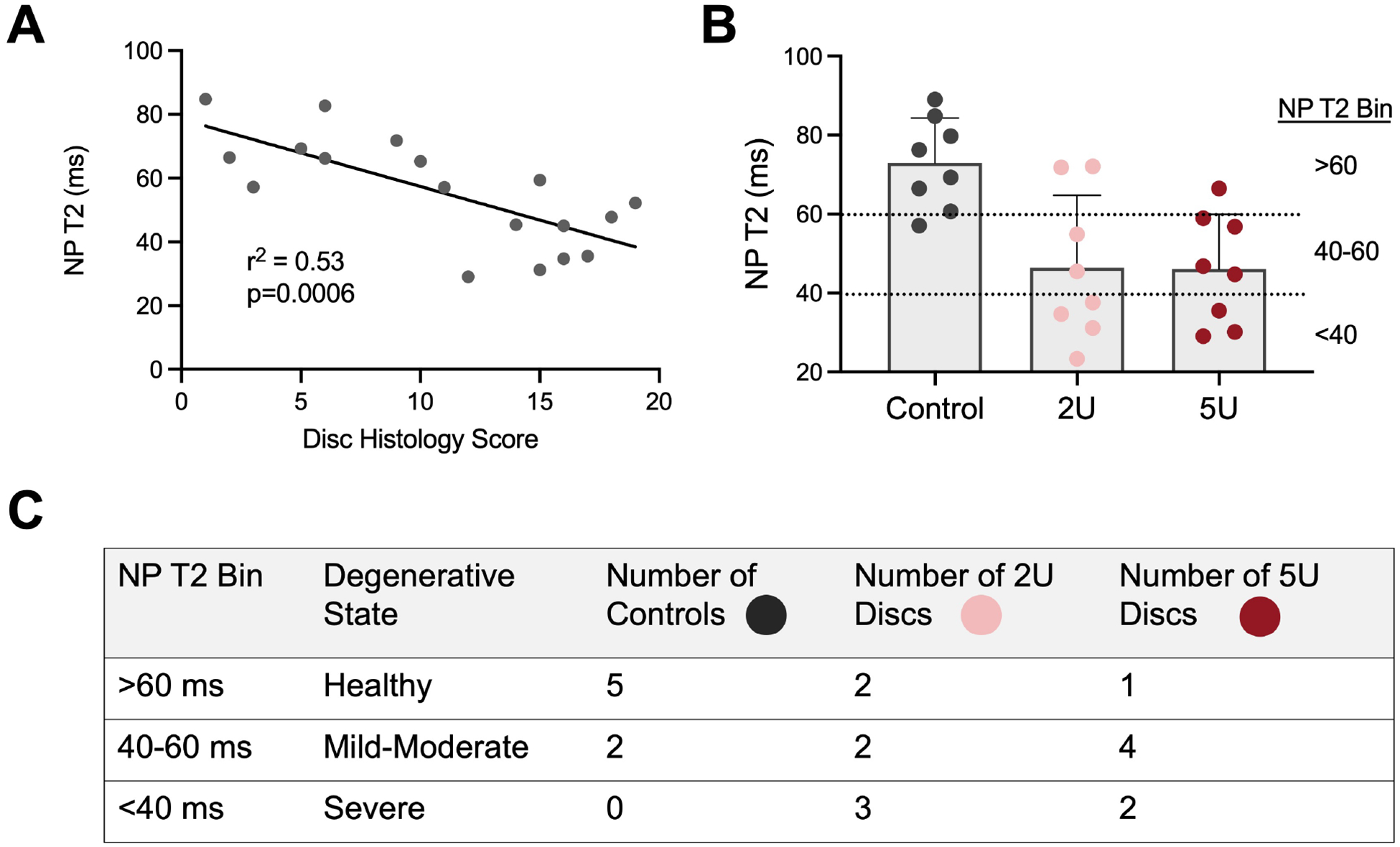
Stratification of samples by Disc NP T2 relaxation time. (**A**) Linear correlation between NP T2 relaxation time and total disc histology score. (**B**) For subsequent analyses, discs were stratified by the designated bins of NP T2 relaxation time due to the similar levels of heterogeneous degeneration achieved by the 2U and 5U doses of ChABC. (**C**) Chart summarizing the number of discs from each experimental group (control, 2U ChABC, 5U ChABC) in each T2 bin. T2 relaxation times >60 ms were designated “healthy”, T2 relaxation times between 40 and 60 ms were designated as “mild-moderate” degeneration, and T2 relaxation times <40 ms were designated as “severe” degeneration.

Given the heterogeneity in degeneration observed within each experimental group, and the absence of statistical differences between ChABC doses with respect to disc height, quantitative MRI and histology scoring outcomes, samples were stratified by severity of disc degeneration (as opposed to experimental group) for subsequent disc, end-plate, and facet analyses. T2 relaxation times within the NP (quantified by MRI T2 mapping) were utilized to stratify samples into three groups of approximately equal sample sizes. MRI T2 mapping provides a sensitive, non-invasive, and clinically translatable measure of disc health. T2 relaxation times significantly correlated with disc histology score in the current study (Fig. 4A) and have been previously shown to correlate with disc water and proteoglycan content, and Pfirrmann grade (Gullbrand *et al*., 2016; Gullbrand *et al*., 2017). Samples were grouped into three degenerative grades (Fig. 4B,C) – ‘healthy’ (T2 > 60 ms, n = 8 discs), ‘mild-moderate’ degeneration (40 ms < T2 < 60 ms, n = 8 discs), and ‘severe’ degeneration (T2 <40 ms, n = 5 discs).

### Intervertebral Disc Compressive Mechanics

**Fig. 5.**
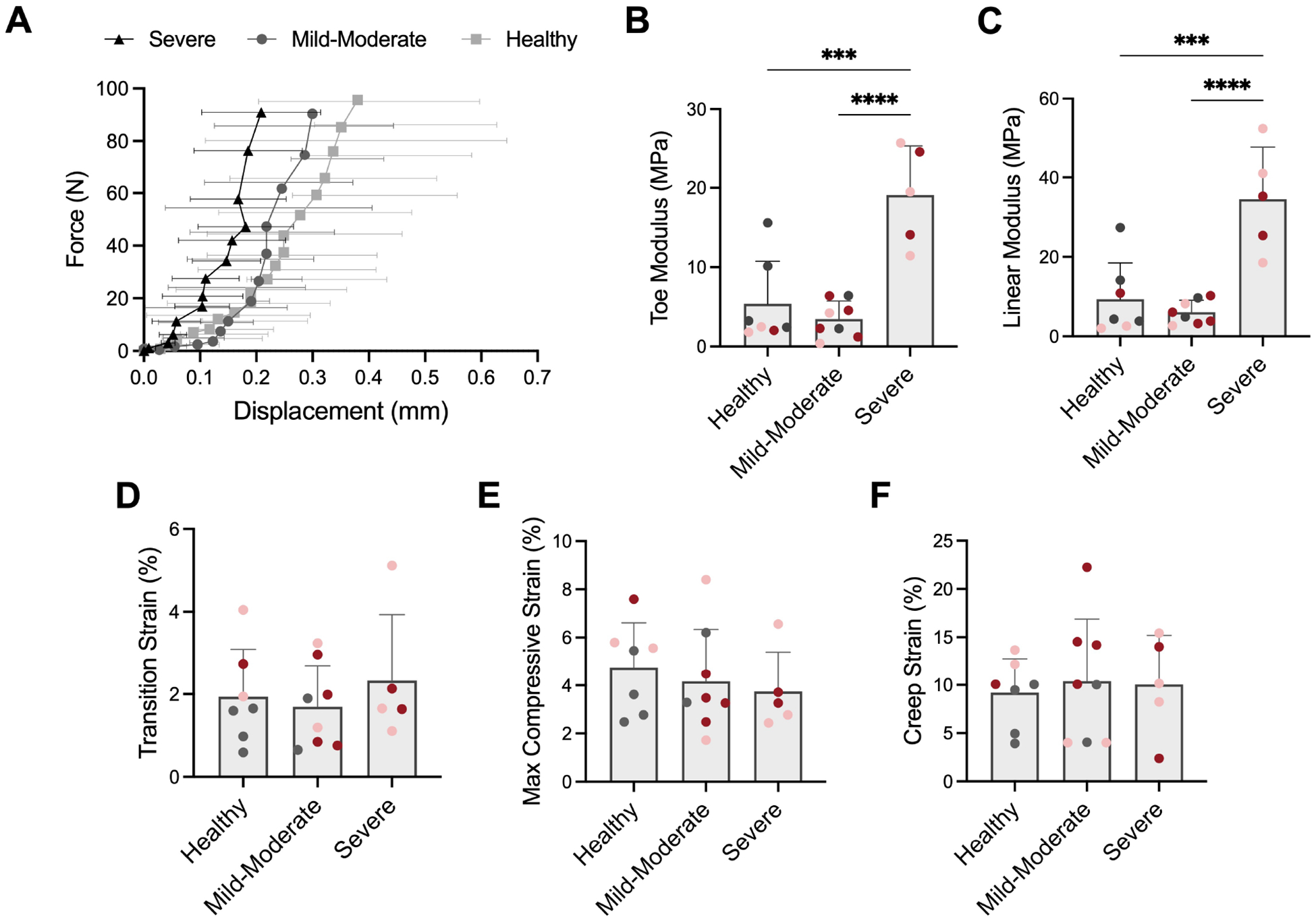
Intervertebral disc mechanics. (**A**) Representative stress strain curves of discs from each degenerative category identified, as indicated in Fig. 4, from which compressive mechanical properties were obtained, including (B) toe region modulus, (**C**) linear region modulus, (**D**) transition strain and (**E**) maximum compressive strain. (**F**) Creep strain was determined from a one-hour creep test in compression. *** = *p* < 0.001, **** = *p* < 0.0001. Coloration of dots denotes experimental group, where grey = control, pink = 2U ChABC and red = 5U ChABC.

Representative stress-strain curves from compressive mechanical testing of the motion segments revealed progressive stiffening of the disc with increasing severity of disc degeneration (Fig. 5A). The toe region modulus of severely degenerative discs significantly increased 250 % and 450 % compared to healthy discs and discs with mild-moderate degeneration, respectively (Fig. 5B). Linear region modulus also significantly increased in severely degenerative discs by 270 % and 470 % compared to healthy discs and discs with mild-moderate degeneration, respectively (Fig. 5C). No detectable differences were observed in transition strain, maximum compressive strain or creep strain across degenerative groups (Fig. 5D–F).

### Trans-Endplate Diffusion and Endplate Pathology

**Fig. 6.**
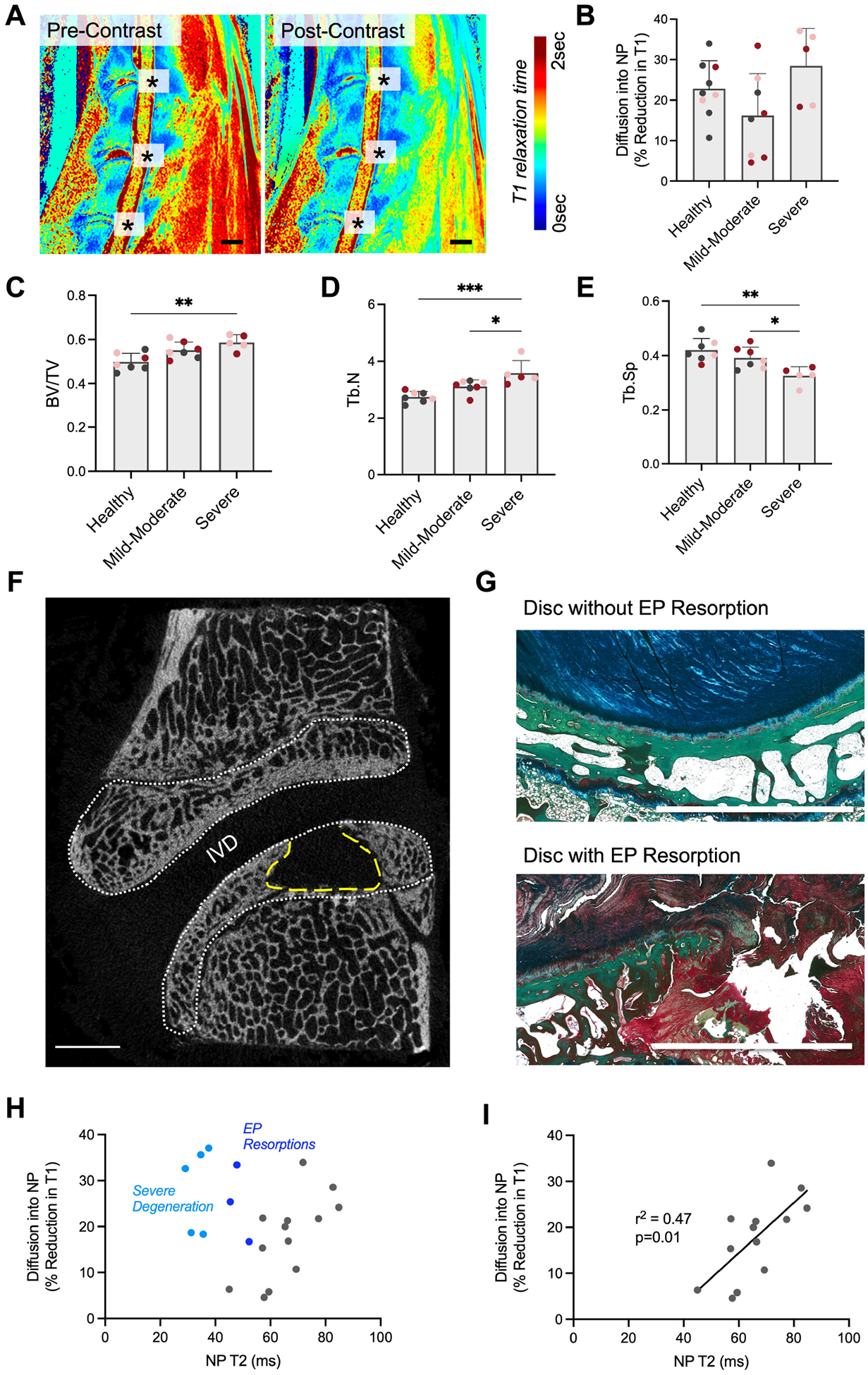
Disc nutrition and endplate pathology. (**A**) Representative T1 relaxation time constant maps of the cervical spine obtained via *in vivo* MRI before and 30 minutes following administration of gadodiamide intravenously, which reduces the T1 relaxation times of the tissues it perfuses. The * denotes the location of each disc. Scale = 20 mm. (**B**) Diffusion of gadodiamide into the disc is quantified as the percent reduction in T1 relaxation time in the NP from the pre- to post- contrast maps. The closer to zero the percent reduction in T1 relaxation time, the less gadodiamide has diffused into the disc. (**C**) μCT quantification of vertebral endplate bone volume fraction (BV/TV), (**D**) Trabecular number (Tb.N) and (**E**) Trabecular spacing (Tb. Sp). * = *p* < 0.05, ** = *p* < 0.01, *** = *p* < 0.001. Coloration of dots denotes experimental group, where grey = control, pink = 2U ChABC and red = 5U ChABC, (**F**) Sagittal μCT slice highlighting a region of endplate resorption in the yellow dashed contour. The ROI analyzed for the bone morphometry parameters is indicated by the white dashed contour. (**G**) Red, green, blue (RGB) stained histology sections demonstrating endplate resorption pathology. Scale = 5 mm. (**H**) Scatterplot of NP T2 relaxation time versus diffusion into the disc, highlighting samples with endplate (EP) resorptions and severe disc degeneration (NP T2 <40 ms). Endplate resorptions were observed in two discs with severe degeneration and three discs with mild-moderate degeneration. (**I**) Linear correlation between NP T2 relaxation time and diffusion into the disc when samples with endplate resorptions and severe disc degeneration were excluded.

To investigate disc-endplate crosstalk in this model, contrast-enhanced MRI with T1 mapping was utilized to measure small molecule diffusion into the disc. The administered MRI contrast agent, gadodiamide, reduces the T1 relaxation time of the tissue proportional to the concentration of contrast agent within the tissue (Fig. 6A), such that diffusion into the NP can be quantified as pre- to post-contrast percent reduction in T1 relaxation time in the NP tissue. Diffusion was highly heterogeneous within each category of disc degeneration, with no detectable differences seen between groups (Fig. 6B). Endplate bone morphometry was next investigated as a potential contributor to altered diffusion into the disc. Endplate bone volume fraction increased with increasing disc degeneration severity, and significantly increased by 17.7 % in the endplates adjacent to severely degenerative discs compared to endplates adjacent to healthy discs (Fig. 6C). This increased bone volume fraction was driven by significant increases in trabecular number (Tb.N) and reductions in trabecular spacing (Tb.Sp), compared to healthy discs (Fig. 6D–E, ROI analyzed denoted by white dotted lines in Fig. 6F). Despite this evidence of endplate sclerosis, areas of endplate bone resorption were observed at some levels in the ChABC groups (Fig. 6F, yellow dashed contour), comprised of unmineralized collagen-rich tissue (Fig. 6G). When all experimental levels were included in a scatter plot of NP T2 relaxation time versus diffusion into the NP, no clear relationship between these two variables was evident (Fig. 6H). However, when samples with either severe disc degeneration (NP T2 < 40 ms) or endplate resorption were excluded, we observed a significant positive linear correlation between NP T2 and diffusion into the NP (Fig. 6I). We justified the removal of these samples from the correlation analyses given that prior work using post-contrasted enhanced MRI in human subjects has demonstrated that transport was enhanced in both severely degenerative discs and discs with endplate defects (Rajasekaran *et al*., 2004).

### Facet Joint Pathology Adjacent to Degenerative Discs

**Fig. 7.**
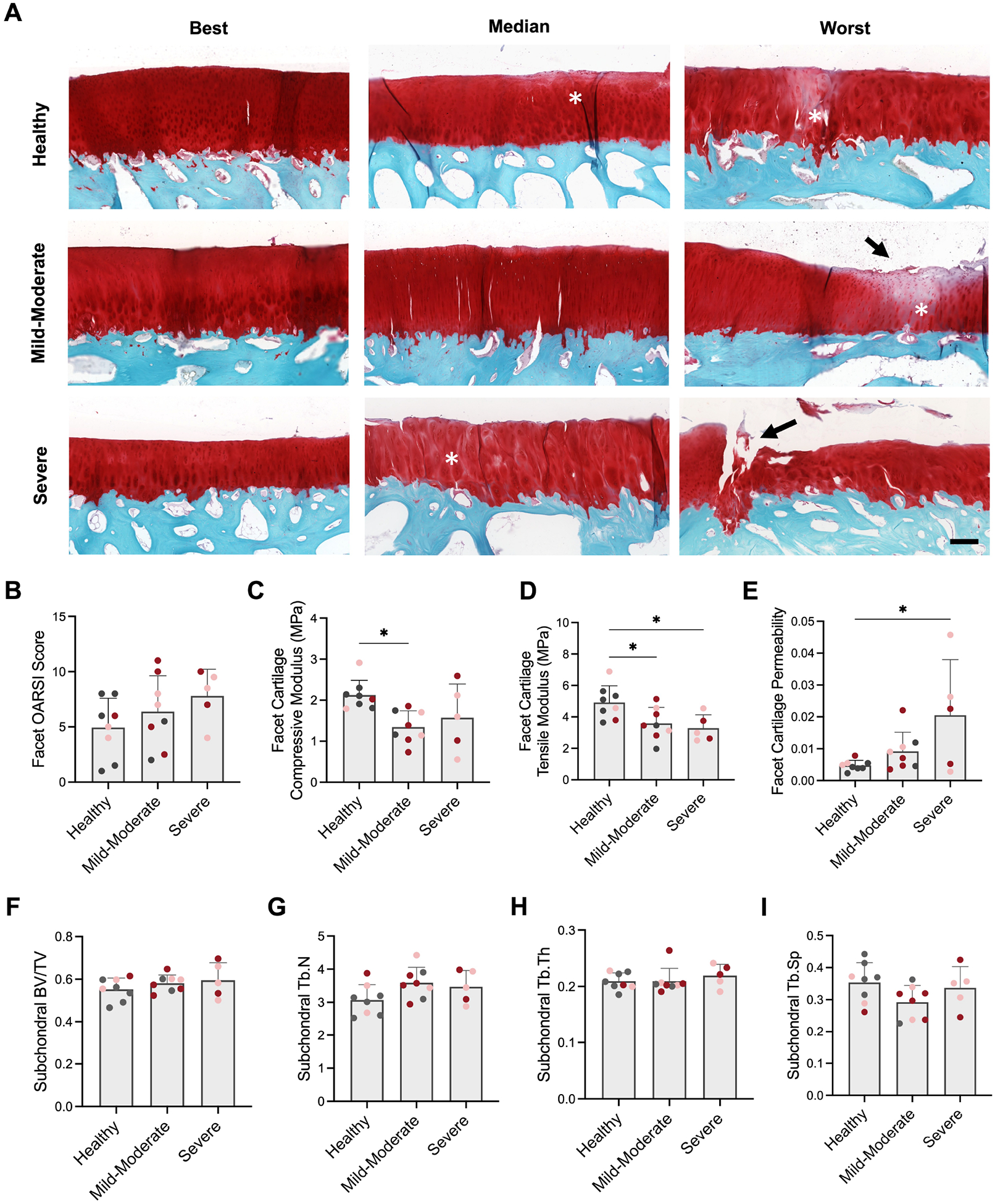
Facet pathology. (**A**) Best (least degenerative), median and worst (most degenerative) sagittal histology of facet articular surfaces adjacent to discs in each degenerative category. Scale = 250 μm, Safranin-O and Fast Green stain. Asterisks indicate regions of reduced proteoglycan staining; arrows indicate regions of cartilage erosion. (**B**) OARSI histopathology score of the facet cartilage. (**C**) Facet cartilage compressive modulus, (**D**) tensile modulus and (**E**) permeability, as measured via creep indentation testing. (**F**) Facet subchondral bone volume fraction (BV/TV), (**G**) trabecular number (Tb.N), (**H**) trabecular thickness (Tb.Th) and (**I**) trabecular spacing (Tb.Sp.). * = *p* < 0.05. Coloration of dots denotes disc experimental group, where grey = control, pink = 2U ChABC and red = 5U ChABC.

Safranin-O and Fast green stained histology sections of the facet articular surfaces paired to each disc across the spectrum of degeneration revealed cartilage pathology, including fibrillation and erosion of the cartilage surface, and reductions in cartilage proteoglycan staining and chondrocyte density, indicative of early-stage osteoarthritis (Fig. 7A). OARSI scores for the facets generally increased with increasing severity of disc degeneration. However, due to the heterogeneity in facet degeneration, statistical differences between degenerative grades were not observed (Fig. 7B). Disc degeneration severity did however have a significant effect on the viscoelastic mechanical properties of the adjacent facet cartilage. Facet cartilage compressive modulus was significantly reduced by 57.8 % adjacent to mild-moderately degenerative discs, while tensile modulus was reduced adjacent to mild-moderately degenerative and severely degenerative discs, compared to healthy discs (Fig. 7C,D). Facet cartilage permeability significantly increased by 330 % adjacent to severely degenerative discs compared to healthy discs (Fig. 7E). There were no detectable differences in facet subchondral bone morphometry amongst disc degeneration grades (Fig. 7F–I).

Finally, a Pearson correlation matrix was utilized to elucidate significant correlations between paired disc and facet structure-function variables (Table 1). Disc NP T2 was significantly and positively correlated with facet cartilage tensile modulus and negatively correlated with facet cartilage permeability. Disc histology score had a significant positive correlation with facet cartilage permeability and a negative correlation with facet cartilage compressive modulus. Disc toe and linear region modulus were both significantly positively correlated with facet cartilage permeability.

## Discussion

Our data demonstrate that the induction of intervertebral disc degeneration in an initially healthy spine can incite degenerative changes across all spine substructures. In this study, degeneration of the intervertebral discs of the goat cervical spine was induced via intradiscal injection of ChABC. This approach has been previously utilized to initiate disc degeneration in the lumbar spine of various species, including goats (Gullbrand *et al*., 2017; Hoogendoorn *et al*., 2007; Zhang *et al*., 2020), sheep (Borem *et al*., 2021), and rabbits (Yang *et al*., 2022). The goat cervical spine is an attractive translational model for the human cervical or lumbar spine due to its semi-upright nature, large disc size and comparable intradiscal loading to the human spine (Agazzi *et al*., 2007; Gilad and Nissan, 1986; Qin *et al*., 2012). Following intradiscal ChABC delivery to goat cervical discs, we observed that disc degeneration progressed in severity over the 12-week study duration, with no signs of spontaneous regeneration or repair of the disc tissues. Disc degeneration in this model was characterized by reduced disc height, reduced disc water and proteoglycan content, disorganization of the annulus fibrosus and loss of cellularity in the nucleus pulposus, mimicking many hallmarks of human disease.

We observed no detectable differences in metrics of disc degeneration between the 2U and 5U doses of ChABC and, consistent with previous studies by our group and others in large animals, there was substantial heterogeneity within each experimental group. Since we achieved similar spectrums of degeneration with both doses of ChABC, we stratified samples by disc health (quantified by T2 relaxation times in the NP) for further analysis to elucidate differences in pathology of the endplates and facets across the spectrum of disc degeneration. With respect to the end-plate, we observed pathologic changes to the cartilage end-plate on histology, including thickness irregularities and focal disruptions. In some discs for each ChABC dose, large areas of subchondral bone resorption were observed, with extrusion of the disc tissue into the lytic subchondral space of the vertebra. These endplate resorptions have been observed in our prior work in the goat lumbar ChABC-induced degeneration model and are similar to Schmorl’s nodes in humans, which are characterized by displacement of disc tissues through the cartilaginous and bony endplates (Gullbrand *et al*., 2017; Mattei and Rehman, 2014; Zehra *et al*., 2017). In humans, Schmorl’s nodes often occur concomitant with disc degeneration. Schmorl’s nodes have been implicated as a contributor to back pain due to the incitement of inflammatory responses within the vertebral marrow space (Heggli *et al*., 2023; Kyere *et al*., 2012). Despite these areas of endplate resorptions, endplate bone density increased with increasing severity of disc degeneration, suggestive of a compensatory sclerotic response in the end-plate adjacent to the resorbed region.

When considering all samples, no detectable differences in trans-endplate, small molecule diffusion into the disc were observed, as measured by post-contrast enhanced MRI T1 mapping. Prior experimental work in human subjects using contrast enhanced MRI has demonstrated that diffusion into the disc was significantly reduced in moderately degenerative discs compared to healthy discs; however, diffusion was increased in both severely degenerative discs and discs with Schmorl’s nodes or endplate defects (Rajasekaran *et al*., 2004). Our results are consistent with these prior findings, with diffusion into the disc increased in the presence of endplate resorptions or severely degenerative discs (NP T2 <40 ms). This result is likely due to increased marrow contact with the disc in the case of Schmorl’s nodes (which we observe to contain fibrovascular tissue), or due to vascular ingrowth into severely degenerative discs. However, vascular infiltration into the disc was not quantified in the current study – further studies are needed to confirm this hypothesis in severely degenerative discs. When samples with endplate resorptions or severe disc degeneration were excluded, we observed a significant positive linear correlation between NP T2 relaxation times and trans-endplate diffusion into the disc, demonstrating that diffusion into degenerative discs is reduced when the boundaries remain intact. The causes of reduced diffusion into the disc with increasing degeneration are likely multifactorial, and likely due to increased endplate bone volume fraction and reductions in vascularity and remodeling of the cartilage endplate – all of which have been shown to contribute to reduced diffusion into the disc (Ashinsky *et al*., 2020; Wong *et al*., 2019).

In addition to degenerative pathology in the boney endplates, we also observed evidence of early-stage osteoarthritic changes in the facet joints posterior to the discs, despite no direct surgical intervention at this location. Only two animal model studies of disc degeneration have assessed concomitant changes to the facet joints. Histologic evidence of facet osteoarthritis was reported adjacent to sheep lumbar discs where degeneration was induced via annular injury (Moore *et al*., 1999). Immobilization of sheep lumbar spinal motion segments via posterior pedicle screw and rod fixation induced not only disc degeneration, but also histologic and radiologic evidence of degeneration of the adjacent facet joints (Wang *et al*., 2018). In our ChABC induced disc degeneration model, we observed significant alterations in facet cartilage mechanical properties without statistically significant structural degenerative facet changes, as quantified by OARSI histopathologic scoring. This finding is in agreement with prior work in a mouse knee destabilization of the medial meniscus (DMM) model of OA, where the modulus of the medial condyle cartilage was significantly reduced as early as 1 week following DMM, whereas histologic signs of OA only became detectable at 4 to 8 weeks post-DMM (Doyran *et al*., 2017).

Facet cartilage mechanical properties were moderately and significantly correlated with measures of disc health, including NP T2 relaxation times, disc histology scores and disc mechanical properties. These correlations highlight the importance of disc-facet crosstalk and, with further work, may inform improved diagnostic methods and treatment approaches for spinal degeneration. In humans, conflicting evidence exists regarding correlations between disc degeneration and adjacent facet OA. Our prior work in human cadaveric samples demonstrated significant correlations between facet OARSI scoring and bone morphometry and measures of adjacent disc mechanics (Gupta *et al*., 2022). Facet cartilage mechanical properties were not correlated with measures of disc degeneration, however, facet OA was significantly more advanced in these human samples compared to the current goat model. Prior work by other groups has demonstrated significant correlations between levels of disc degeneration and facet OA on clinical imaging (Mesregah *et al*., 2020; Song *et al*., 2019), yet other studies have suggested no correlation between disc and facet pathology measured histologically (Gries *et al*., 2000). While we did not measure loading in the facet joints directly in this study, we hypothesize that the early-stage OA observed in the facet joints in the goat model is caused by aberrant spine biomechanics, wherein altered disc mechanical properties (due to a loss of disc hydration and proteoglycan content) contribute to aberrant loading of the adjacent facet joints (Lu *et al*., 2020). In humans, it has been suggested that the portion of axial load borne by the facet joints increases with advanced degeneration that includes a severe loss of disc height (Li *et al*., 2011). This is consistent with the well-established contribution of aberrant loading in other synovial joints such as the knee (usually due to disruption of either the ACL or meniscus) in the initiation and progression of OA (Felson, 2013).

In conclusion, in this study, we established a large animal model of disc degeneration featuring pathology and crosstalk in all spine substructures, with this pathology pathognomonic to the human degeneration in the disc, end-plates and facet joints. However, this study is not without limitations. While many similarities exist between goat cervical spine anatomy and human cervical or lumbar spine anatomy, differences do exist in facet joint surface inclination between the two species, which likely affects load sharing between the disc and facet and the mechanisms by which altered disc mechanics contributes to facet OA. (Wilke *et al*., 1997b; Wilke *et al*., 1997a) These anatomical and biomechanical differences are more pronounced when comparing the goat cervical spine to the human lumbar spine, and thus further research is needed to validate the applicability of this model to the human lumbar spine. We also observed significant variability in not only the response of the experimental discs to ChABC injection, but also in the health of the adjacent native discs that did not receive ChABC injection. This finding was a primary motivator for stratifying by disc health rather than by experimental group for further analyses. However, depending on the future use of the model and study goals, adjacent discs may be less than ideal as a ‘healthy’ control. Despite this variability, we achieved robust effect sizes (>0.6) for many of our outcome variables, requiring sample sizes between 3 and 9 per group to yield a power of 0.8. ChABC dose or stratification by disc health had a negligible effect on other variables (disc compressive strain, facet OARSI score, facet bone volume fraction and %T1 reduction), with small effect sizes (<0.25) that would require infeasible sample sizes (22–84 samples per group) to yield adequate power. In the future, use of alternative outcome metrics, such as multi-axial disc mechanical testing using torsion or bending modalities may prove more sensitive to the structure-function changes occurring with spinal degeneration. Future work will also focus on investigating biologic contributors (such as inflammation, neovascularization and neoinnervation, and immune cell infiltration) to the progression of spinal disease in this model, in addition to paraspinal muscle degeneration, which is frequently observed concomitant with back pain and disc degeneration in humans (Khattab *et al*., 2022). As human spinal degeneration rarely manifests in a single compartment of the spine, this model may increase the translational relevance of studies attempting to assess the efficacy of novel disc repair or regeneration strategies.

## Figures and Tables

**Table 1.** Statistically significant Pearson correlations between quantitative measures of disc and facet health.

Disc Parameter	Facet Parameter	Pearson r	*p*-value
**NP T2**	Cartilage Tensile Modulus	0.554	0.010
	Cartilage Permeability	−0.547	0.009
**Disc Histology Score**	Cartilage Permeability	0.543	0.020
	Cartilage Compressive Modulus	−0.502	0.034
**Disc Linear Modulus**	Cartilage Permeability	0.685	0.001
**Disc Toe Modulus**	Cartilage Permeability	0.569	0.009

## Data Availability

All datasets are included in the published manuscript.

## List of Abbreviations

ChABC: chondroitinase ABC
CEP: cartilage end-plates
NP: nucleus pulposus
AF: annulus fibrosus
MRI: magnetic resonance imaging
OA: Osteoarthritis
IV: intravenous
IM: intramuscular
PBS: sterile phosphate buffered saline
DHI: disc height index
Tb.N: trabecular number
Tb.Sp: trabecular spacing
EP: endplate
Tb.Th: trabecular thickness
